# Phosphoregulation of Ire1 RNase splicing activity

**DOI:** 10.1038/ncomms4554

**Published:** 2014-04-07

**Authors:** Filippo Prischi, Piotr R. Nowak, Marta Carrara, Maruf M. U. Ali

**Affiliations:** 1 Department of Life Sciences, Centre for Structural Biology, Sir Ernst Chain Building, Imperial College London, London SW7 2AZ, UK

## Abstract

Ire1 is activated in response to accumulation of misfolded proteins within the endoplasmic reticulum as part of the unfolded protein response (UPR). It is a unique enzyme, possessing both kinase and RNase activity that is required for specific splicing of Xbp1 mRNA leading to UPR activation. How phosphorylation impacts on the Ire1 splicing activity is unclear. In this study, we isolate distinct phosphorylated species of Ire1 and assess their effects on RNase splicing both *in vitro* and *in vivo*. We find that phosphorylation within the kinase activation loop significantly increases RNase splicing *in vitro.* Correspondingly, mutants of Ire1 that cannot be phosphorylated on the activation loop show decreased specific Xbp1 and promiscuous RNase splicing activity relative to wild-type Ire1 in cells. These data couple the kinase phosphorylation reaction to the activation state of the RNase, suggesting that phosphorylation of the activation loop is an important step in Ire1-mediated UPR activation.

## Abstract (web summary)

Ire1 is an effector of the unfolded protein response that is activated upon stress to maintain protein homeostasis. Here, Prischi *et al*. demonstrate that phosphorylation of Ire1 within its kinase activation loop increases its RNAse activity, thus identifying a regulatory cross-talk between the two domains.

## Graphical abstract

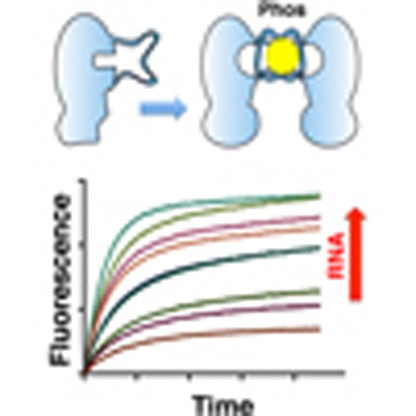

The endoplasmic reticulum (ER) is the site of synthesis, modification and folding for membrane proteins and secretory proteins destined for other cellular compartments and the extracellular space. Upon ER stress, the influx of nascent polypeptides overwhelms the capacity of the ER to correctly fold and maintain protein tertiary structure, and, as a result, a complex cell signalling system termed the Unfolded Protein Response (UPR) is activated to maintain homeostasis. The UPR elicits a varied response, which includes transcriptional upregulation of chaperones, general cell translation attenuation, activation of ER-associated degradation (ERAD) and an increase in ER size. If the imbalance is not rectified, then the UPR switches from being prosurvival to instigating an apoptotic response^1^,^2^.

Ire1 is one of three sensor proteins that initiates and ultimately dictates the outcome of the response in humans. Ire1 consists of a luminal domain, a single pass transmembrane segment and a cytoplasmic domain that is further subdivided into an autophosphorylating kinase domain and an endoribonuclease (RNase) domain.

Upon activation, the luminal domains of Ire1 dimerize/oligomerize; this positions the cytoplasmic kinase domains in close proximity to each other in a face-to-face orientation, which allows autophosphorylation to occur^3^. On the basis of yeast structures, Ire1 reorientates to form a back-to-back rearrangement, which may form higher oligomeric structures, and is thought to be important to achieve the RNase splicing competent state^4^,^5^.

In budding yeast, RNase domain splices a 252-nucleotide segment specific to HAC1 mRNA in a spliceosome-independent manner. The 3′ untranslated region of HAC1 contains a targeting sequence, which may be required for mRNA recruitment to the oligomerized Ire1 active state. By contrast, in the human system, unspliced Xbp1 contains a C-terminal signal sequence that recruits the ribosome-nascent mRNA chain to the ER membrane to undergo splicing by Ire1 (refs 1, 6). Ire1 splices a shorter 26-nucleotide sequence from Xbp1 mRNA. This causes a translational frameshift, which results in the expression of a potent transcriptional activator that upregulates expression of UPR target genes^2^,^7^,^8^.

UPR has been implicated in many diseased states, notably cancer. Interestingly, Ire1 has recently emerged as a new target for therapeutic intervention in multiple myeloma (MM), a cancer resulting from malignant transformation of plasma cells. Xbp1, besides its role in UPR, acts as a checkpoint control in plasma cell differentiation, and misregulation can cause uncontrollable proliferation leading to MM^9^,^10^,^11^. We have previously demonstrated that inhibiting the human Ire1 kinase reaction with a specific kinase inhibitor sunitinib resulted in loss of Xbp1 splicing in MM cell lines^3^.

Mechanistic understanding of how phosphorylation and ligand binding affect the RNase activity of Ire1 is of crucial importance for therapeutic interventions. Recently, phosphomimetic mutants of yeast Ire1 were seen to undergo sustained splicing, indicating that dephosphorylation of Ire1 is an important step in RNase deactivation^12^. However, a separate study suggested that the phosphoryl transfer reaction is important for deactivation rather than activation of RNase^13^, which supports a previous work suggesting that the kinase inhibitor 1NM-PP1 activated RNase splicing thereby circumventing the requirement for phosphorylation^1^,^14^. This contrasts with early experimental work showing mutation of catalytic residues in the kinase domain disrupting RNase function^15^ and more recently inhibition of kinase activity leading to loss of splicing *in vivo* 3,15,16. Furthermore, sunitinib has also been shown to be a potent inhibitor of closely related RNase L and PKR; these proteins are involved in antiviral innate immune responses and share significant sequence homology with Ire1 kinase^17^.

In an attempt to clarify the role of phosphorylation and ligand binding upon the RNase splicing activity of Ire1, we identify and map, using mass spectrometry analysis, specific phosphorylation sites by purifying distinct phosphorylated Ire1 populations from proteins expressed in the eukaryotic insect cell system. The isolation of differentially phosphorylated species allows us to assay the effects of specific phosphorylations upon the RNase splicing activity. We show that phosphorylations upon the activation loop increase the enzymatic rate of splicing manyfold above that of the dephosphorylated protein *in vitro*, whereas linker region and RNase domain phosphorylations have little/no impact upon splicing. Trans-autophosphorylation of dephosphorylated Ire1 *in vitro* leads to phosphorylation of two specific residues in the kinase activation loop. C-terminal Ire1 expressed in insect cells exhibits further phosphorylations that occur on the Linker and RNase domain. Furthermore, we show that mutations of activation loop phosphorylation sites result in loss of splicing and a reduction in RIDD activity relative to wild-type Ire1 in cells. This work indicates the importance of the kinase auto-transphosphorylation reaction in activating the RNase domain to achieve the splicing active state, thereby coupling the two reactions together. Phosphorylation of kinase activation loop is an important and necessary step for achieving the activated Ire1 RNase splicing state in which splicing of Xbp1 is enhanced, leading to subsequent activation of UPR signal. This study will help to clarify our understanding of Ire1-phosphoregulated RNase splicing and provide valuable insights for cancer therapeutic targeting of Ire1.

## Results

### Identification of phosphorylation sites

**Figure 1:**
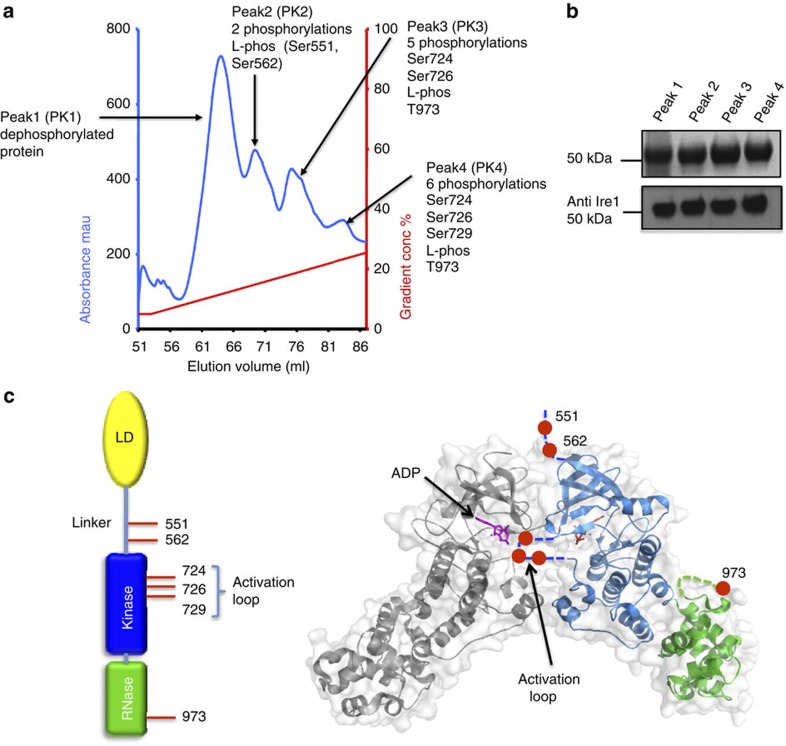
Identification of phosphorylation sites. Chromatogram showing the elution profile of human Ire1 protein (547–977) expressed in insect cells and passed through a monoQ anion exchange column. The four peaks indicated distinct phosphorylated species of Ire1, and their sites of phosphorylation were identified by mass spectrometry (see Supplementary Figs 1,2, Supplementary Table 1). (**b**) Ire1 protein (547–977) samples from the four distinct peaks isolated by anion exchange were visualized by SDS-PAGE (top panel) and by western blot using a generic human Ire1 antibody (bottom panel). (**c**) The position of phospho sites relative to each other and mapped onto the X-ray structure of human Ire1 autophosphorylation complex (3P23).

A cytoplasmic portion of human Ire1 (547–977) encompassing the kinase and endoribonuclease domains was expressed in insect cells. We have previously detailed our purification protocol for obtaining homogenous samples of dephosphorylated protein^3^. In the present protein purification, we omitted the incubation with lambda phosphatase and performed the monoQ anion exchange step with a very shallow gradient (Fig. 1a,b). From this purification step we were able to isolate several different peaks, which we sent for mass spectrometry analysis, where proteins were treated with trypsin digestion. The resultant peptide fragments were subjected to tandem mass spectrometry using MALDI-TOF/TOF and then ESI QTOF instruments (Supplementary Figs 1A–D and 2A,B and Supplementary Table 1). Several peptides exhibited modifications consistent with being phosphorylated. Using the ESI QTOF setup, we were able to map the sites of certain phosphorylations. To further reinforce our data, we analysed the molecular mass of the different phosphorylated protein samples using the MALDI-TOF/TOF setup (Supplementary Fig. 3). Using both trypsin-digested analysis and molecular mass spectrometry of protein peak samples, coupled with the fact that this is an established technique for purifying phosphorylated proteins, particularly kinases^18^,^19^, we were fully confident of the phosphorylated protein assignments. The order of peaks that eluted off from the monoQ column roughly correlated with increasing number of phosphorylations upon Ire1. The first peak (Fig. 1a,b), which was also the largest peak, relates to purified Ire1 protein being in a dephoshorylated state, interestingly, suggesting that Ire1 protein is being kept in a ‘ground dephosphorylated state’ by various phosphatases consistent with other kinase regulatory mechanisms. The next peak (PK2) (Fig. 1a,b) indicated purified protein being phosphorylated at two sites ser551 and ser562 in the linker region; we refer to this as L-phos. Peak 3 (PK3) contained five phosphorylations at ser724, ser726, and L-phos, with a weak/low signal for thr973 phosphorylation. PK4 indicates Ire1 protein phosphorylated at ser724, ser726, ser729 and L-phos, and, again as with PK3, weak/low levels of thr973 phosphorylation were detected. The sites of phosphorylations fall into three particular areas, phosphorylation in the linker region (L-phos), kinase activation loop phosphorylations and thr973 phosphorylation positioned within the C-terminal RNase domain (Fig. 1c). The isolation of differentially phosphorylated Ire1 subspecies allows us to assay the effects of specific phosphorylations upon Ire1 RNA splicing activity; however, initially it is also necessary to understand the effects of ligand binding upon the splicing reaction.

### Ligand binding has minimal effect on RNase activity *in vitro*

**Figure 2:**
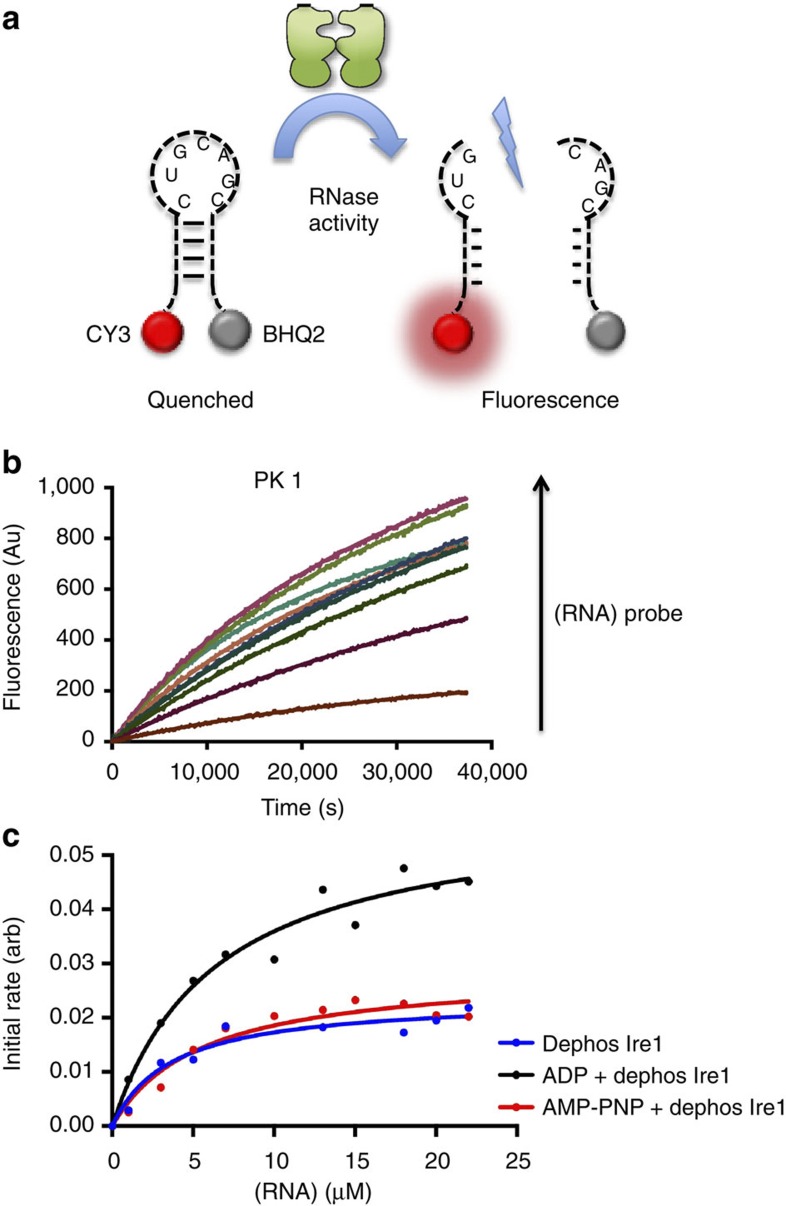
Ligand binding to dephosphorylated Ire1 has little impact on RNase splicing. (**a**) Fret-quenched RNA Xbp1 substrate probe was tagged with Cy3 label at the 5′ end and with BHQ2 quencher at 3′ end. The substrate probe was cleaved by Ire1 allowing measurement of fluorescence emission at 590 nm. (**b**) Fluorescence emission time course experiment measuring RNase splicing of FRET probe by Ire1 *in vitro*. Dephosphorylated Ire1 protein was incubated with substrate probe, and cleavage reaction was followed by fluorescence emission over a period of time. The time course was repeated with varying concentrations of the substrate probe. All experiments were performed in triplicate. (**c**) The initial rates of reactions calculated from each profile obtained from fluorescence time course splicing experiment in **b** were plotted against various concentrations of substrate probe to give initial rate curves that obeyed Michelis–Menton kinetics. The initial rate curves for both dephosphorylated Ire1 (blue) and dephosphorylated Ire1 bound to AMP-PNP (red) gave similar profiles, whereas dephosphorylated Ire1 bound to ADP (black) gave a slightly elevated profile (see Table 1).

To assay the effects of phosphorylations and ligand binding upon RNase splicing activity, we made use of fluorescence resonance energy transfer (FRET)—quenched mini Xbp1 RNA substrate probe (Fig. 2a), which when cleaved by Ire1 emits fluorescence at 590 nm (cy3) wavelength^20^. Initially, just taking the dephosphorylated protein, we incubated varying concentrations of substrate probe and measured the fluorescence emitted over a period of time (Fig. 2b). The resultant reaction data were of very high quality and the reactions fitted excellently with Michaelis–Menton enzyme kinetics. By using the initial rates of each reaction at varying concentrations of the probe, we were able to successfully measure the substrate turnover (*K*_cat_) and the catalytic enzyme efficiency (*K*_cat_/*K*_m_) (Table 1). We performed the same analysis for both dephosphorylated proteins in the presence of a non-hydrolysable ATP mimic AMP-PNP and ADP (Fig. 2c). We found that the *K*_cat_ values for both the dephosphorylated and the AMP-PNP bound cofactors were almost identical at 0.118 and 0.144 s^−1^; this was also reflected in the *k*_cat_/*K*_m_, which gives a measure of the efficiency of the enzyme, at 3.21 × 10^4^ and 2.63 × 10^4^ M^−1^ s^−1^, respectively. For dephoshorylated protein in the presence of ADP, the *K*_cat_ and *K*_cat_/*K*_m_ were only slightly higher at 0.295 s^−1^ and 4.61 × 10^4^ M^−1^s^−1^, suggesting that the bound ADP may assist in a conformation conducive for the RNase cleavage reaction. Previously reported data in the field suggested that the presence of nucleotide can induce RNase activity; if this were the case, then AMP-PNP would also have an inducing effect, which in our data it does not and is instead clearly identical to the basal level splicing of dephosphorylated protein without any ligand, suggesting that just the mere presence of nucleotide is not enough to induce significant splicing. However, on comparison of the dephosphorylated Ire1 rates of reaction with those of the phosphorylated Ire1 protein (see below PK3 and PK4), the values between dephosphorylated, ADP and AMP-PNP bound protein showed insignificant differences.

### Activation loop phosphorylation increases RNase activity

**Figure 3:**
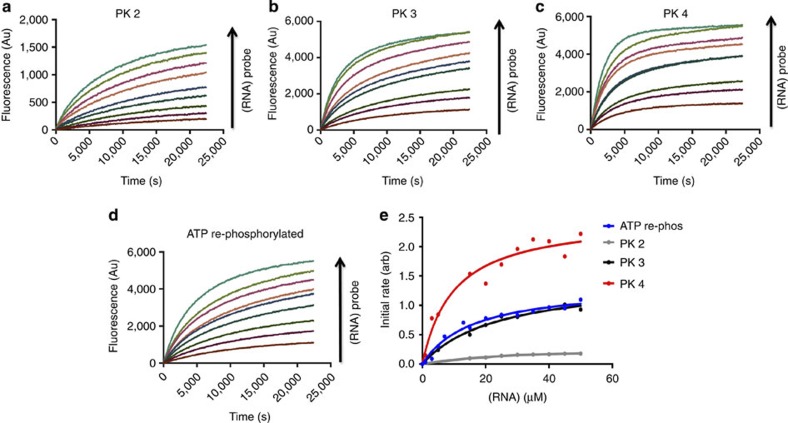
Distinct phosphorylations significantly increase RNase splicing. (**a**–**d**) Fluorescence time course experiments measuring splicing of FRET probe by differently phosphorylated species of Ire1 protein at increasing concentrations of substrate probe. (**a**) Profile of PK 2 Ire1 protein. (**b**) Profile of *in vitro* ATP re-phosphorylated Ire1 that was initially dephosphorylated. (**c**) Profile of PK 3 Ire1 protein. (**d**) Profile of PK 4 Ire1 protein. (**e**) A plot of the initial rates derived from each fluorescence time course splicing profile in **a**–**d** at varying substrate concentrations to give an overall initial rate curve for that particular protein peak sample from which we obtain the kinetic parameters listed in Table 1. PK 4 Ire1 sample (red) displays the steepest initial rate curve profile, and this is reflected in its kinetic rates *K*_m_ and *K*_cat_ (see Table 1). PK 3 human Ire1 protein sample (black) purified from insect cells with distinct phosphorylations on ser724 and ser726 within the activation loop and with extra L-phos and pT973 gives a very similar initial rate profile to dephosphoshorylated protein that has been re-phosphorylated by incubating with ATP *in vitro* and exhibiting phosphorylations on Ser724 and Ser726 on activation loop only. PK 2 Ire1 protein sample (grey) containing only L-phos modification shows a very slow initial rate curve profile.

The purification and isolation of the differentially phosphorylated Ire1 enabled us to measure the effects of specific phosphorylations upon the RNase activity (Fig. 3a–e). PK2 contains doubly phosphorylated protein in the linker region at ser551 and ser562. Analysis of the initial rate curve for PK2-phosphorylated Ire1 (Fig. 3a,e and Table 1) gave a *K*_cat_/*K*_m_ value of 5.11 × 10^4^ M^−1^ s^−1^. This value is only slightly higher than that of dephosphorylated Ire1 protein with ADP binding, suggesting that phosphorylations within the linker region do not have much of an effect on the catalytic efficiency of Ire1 splicing reactions, which is not that surprising as it is quite a distance away from the RNase and indeed kinase active sites.

PK3 contains Ire1 protein, which is phosphorylated on L-phos, and three further sites, ser724 and ser726 on the activation loop of the kinase domain and low levels of pthr973 in the RNase domain. Using the FRET assay, we obtained a *K*_cat_ value of 7.37 s^−1^ and *K*_cat_/*K*_m_ of 3.02 × 10^5^ M^−1^ s^−1^ (Fig. 3b,e and Table 1). These values represent a huge increase over the dephosphorylated protein or indeed any of the previous samples tested. The substrate turnover (*K*_cat_) was over 60-fold higher than that for the dephosphorylated protein and at least 24-fold higher than that for dephosphorylated Ire1 with ADP bound. These increases were also mirrored in the *K*_cat_/*K*_m_ values with PK3 Ire1 being an order of magnitude higher than that of dephosphorylated protein. The difference between PK2 and PK3 is essentially of the two phosphorylations, ser724, ser726 (with low level thr973 detected), on the activation loop. These two phosphorylations are responsible for the greatest increase in enzyme RNase activity, even more so than that of PK3 to PK4 (see below PK4). The phosphorylations no doubt have an effect on the conformation of the activation loop and possibly lock it into a position different from that of the dephosphorylated protein. These movements would transmit down to the RNase domain thus allowing it to adopt a conformation, which is more conducive for splicing to occur.

PK4 Ire1 differs from PK3 protein by having one additional phosphorylation positioned on the activation loop at ser729. Using the FRET assay, we obtained the *K*_cat_ value of 12.7 s^−1^ and *K*_cat_/*K*_m_ value of 1.26 × 10^6^ M^−1^ s^−1^ (Fig. 3c,e and Table 1). This represents an increase of more than 105-fold in substrate turnover compared with dephoshorylated protein and almost double that of PK3 Ire1. Again, this is reflected in the catalytic efficiency of *K*_cat_/*K*_m_, which is almost 40-fold higher than that of dephosphorylated protein and over 4-fold higher than that of PK3 Ire1. Although the enzymatic rate values for PK4 are the highest for all samples tested, the greatest increase between samples is that of PK2 to PK3 and is attributable to pS274 and pS726.

It is clear from the enzymatic rate analysis that the phosphorylations on the activation loop enhance the RNase splicing activity of Ire1 manyfold above that of the dephosphorylated protein; in the case where all three of the activation loop residues, ser724, ser726 and ser729, are phosphorylated, the substrate turnover is over 100-fold higher than that of the dephosphorylated protein. This unambiguously links the phosphorylations upon the activation loop to enhanced RNase splicing of Ire1. The phosphorylations upon the linker region and the RNase domain do not seem to effect the splicing reaction. Similarly, the addition of nucleotide ADP and AMP-PNP to dephosphorylated protein seems to have a minimal effect upon the splicing reaction when compared with activation loop phosphorylations. It would be interesting to see how the binding of ligands to the distinct phosphorylated species effects the RNase activity. To test this point, we incubated both ADP and AMP-PNP to the different Ire1 peaks and observed the enzymatic activity (Supplementary Fig. 4A–C). We see that, in the presence of ADP and AMP-PMP, the enzymatic reaction is largely reduced when compared with that of phosphorylated protein without ligand. This indicates that ADP and AMP-PMP, rather than having an activating effect upon splicing, reduce the rate of reaction significantly. Ligand binding possibly causes the glycine-rich loop to close over the ligand-bound active site, locking the kinase domain in a ‘closed’ conformation, with a subsequent conformational change transmitted to RNase domain and negatively effecting the splicing activity.

### *In vitro* Ire1 is re-phosphorylated on ser724 and ser726

The distinct phosphorylated populations of Ire1 purified from insect cells, which contain phosphatases, indicate that they are biologically relevant. It would be interesting to investigate which of these phosphorylations occur solely due to Ire1 kinase auto-transphosphorylation reaction. To test this point, we incubated dephosphorylated protein in the presence of ATP and then subjected the protein to trypsin digestion and subsequent mass spectrometry analysis. The mass spectrometry analysis indicated the presence of two phosphorylations at ser724 and ser726 on the activation loop of the kinase domain. We then tested the substrate turnover and catalytic efficiency by using the FRET probe assay (Fig. 3d,e and Table 1). The *K*_cat_ and *K*_cat_/*K*_m_ values for the *in vitro* re-phosphorylated Ire1 wwere 6.79 s^−1^ and 4.2 × 10^5^ M^−1^ s^−1^, respectively. These values match very closely with those of PK3 Ire1, which was also phosphorylated on ser724 and ser726 of the activation loop with further phosphorylations on L-phos and Thr973, but as L-phos and pThr973 do not contribute significantly to the splicing reaction, we would expect that PK3 and re-phosphorylated protein to have similar values for their enzymatic rates, which is indeed the case. This further supports the notion that L-phos and pThr973 do not contribute greatly to splicing reaction kinetics, and the major factor in the increase in RNase activity is due to phosphorylation of activation loop residues ser724, ser726 and ser729 with the greatest contribution from ser724 and ser726.

### Activation loop mutant reduces RNase splicing in cells

**Figure 4:**
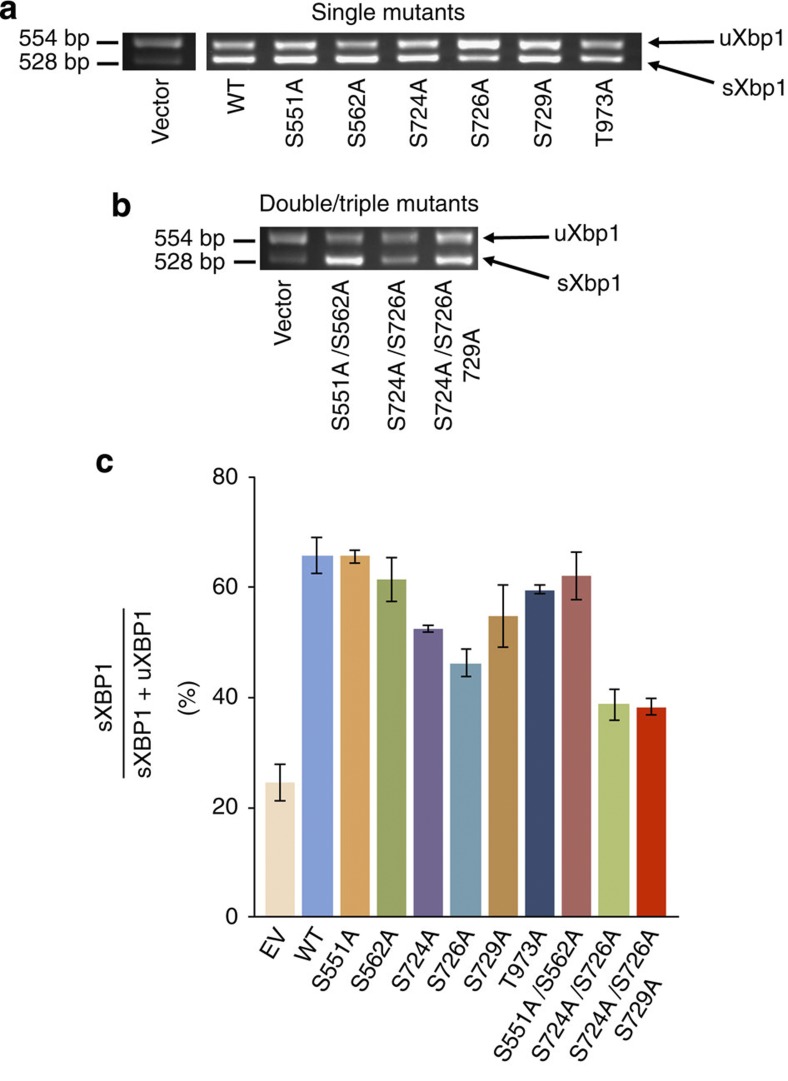
Mutation of phosphorylation sites inhibits splicing in cells. (**a**) Xbp1 splicing in HT 1080 human cell line. Individual phosphorylation mutants were co-transfected into HT1080 cells and subjected to tunicamycin for 2 h to induce ER stress. RT–PCR of sXbp1 and uXbp1 was run on 3% agarose gel and visualized by gel red DNA stain. Empty vector was used as control, and all experiments were repeated in triplicate. (**b**) Xbp1 splicing using sets of mutations mimicking groups of phosphorylations within Ire1. S551A/S562A represents L-phos mutations, S724A/S726A replicates *in vitro* re-phosphorylation of dephos Ire1 with ATP, S724A/S726A/S729A triple mutant represents all activation loop phosphorylations. (**c**) Quantification of inhibition of Xbp1 splicing *in vivo* of each individual phosphorylation mutant and sets of mutants in HT1080 cells, with empty vector (EV) as control. Splicing is clearly reduced in all activation loop mutants and especially in the double and triple mutants recapitulating the *in vitro* findings. Linker region or L-phos single and double mutants seem not to have an impact on splicing, whereas T973A seems to have a small impact. Error bars represent mean±s.e.m., *n*=3.

It is clear that activation loop phosphorylations have a significant effect upon the RNase activity *in vitro*. We wanted to investigate the effects of mutating specific phosphorylations on RNase splicing *in vivo*. To address this point, we co-transfected HT1080 cells with plasmids expressing Ire1 phosphorylation mutations (Fig. 4a–c, Supplementary Fig. 5), identified from our mass spectrometry data, and measured splicing by RT–PCR analysis. Initially, we mutated each phosphorylation site to assay the effect of that specific phosphorylation upon splicing and then used the double and triple mutations to mimic sets of phosphorylations such as L-phos (S551A, S562A), *in vitro* re-phosphorylated activation loop (S724A, S726A) and full activation loop phosphorylations (S724A, S726A, S729A) to establish their effects. Using 50 μM tunicamycin to induce ER stress, we measured the total level of splicing for each mutant and compared it with both wild-type Ire1 and empty vector control. After 2 h, the most drastic effect by a single-amino-acid mutation on splicing was observed with activation loop mutants, S724A, S726A, S729A and, in particular, S726A (Fig. 4a–c), which displayed a 50% reduction in splicing when compared to wild type. This is in agreement with the *in vitro* splicing activity where the biggest effect was seen with PK3, which contains both S724 and S726. The linker region mutations S551A and S562A seem to have very little effect upon splicing; this is further confirmed by the double linker region mutation S551A/S562A, which also did not exhibit significant loss of splicing compared with activation loop mutations. The T973A mutation displayed a slight inhibition of splicing of around 85% of wild type, which was more than L-phos double mutant, but again when compared with the activation loop mutants it becomes less significant. The phosphorylation on residues pS724 and pS726, which is present in PK3 and in *in vitro* re-phosphorylated Ire1, was seen from our FRET assay to have the largest increase in activity (Table 1) corresponding to a fivefold jump from PK2 to PK3, whereas PK3 to PK4 represented a twofold increase in terms of *K*_cat_ values. It is therefore straightforward to predict that the double-mutant S724A/S726A would have a drastic effect and indeed this was the case with splicing *in vivo* reduced to 38%, recapitulating the results obtained *in vitro*. Interestingly, the triple activation loop mutant S724A, S726A, S729A seems to be inhibiting to a similar level as that of the double-mutant S724A, S726A. This indicates that there maybe a basal level of splicing, which is not directly influenced by phosphorylation, and that mutation of S724A and S726A is sufficient to reach this basal level *in vivo*.

Therefore, these data clearly indicate that mutation of activation loop phosphorylation sites upon Ire1 in HT1080 cells significantly reduces RNase splicing, consistent with the *in vitro* FRET splicing data.

### Activation loop mutant reduces splicing in Ire1−/− cells

**Figure 5:**
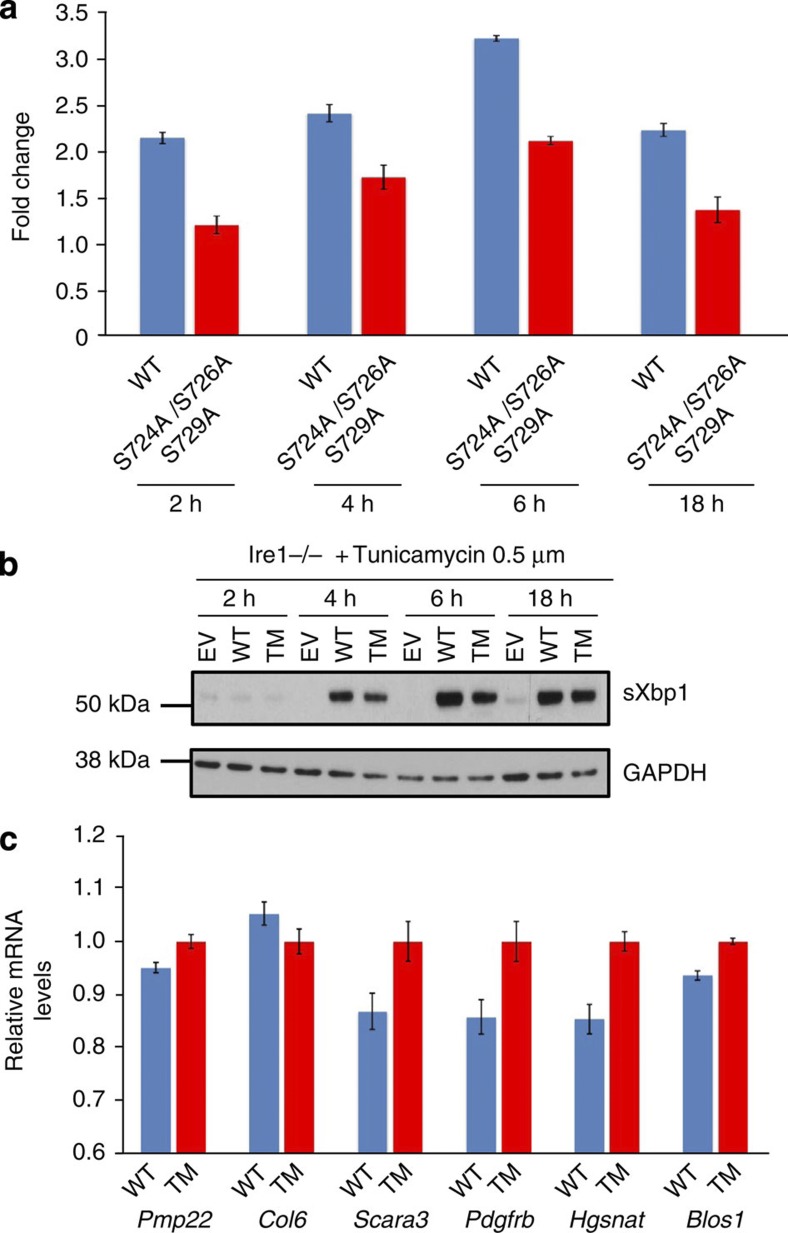
Activation loop phosphorylation mutants retard splicing and RIDD activity in Ire1−/−cells. (**a**) Ire1−/− cells were co-transfected with plasmids containing Ire1 wild-type (WT), triple activation mutant (TM) or empty vector with plasmid containing GFP. Cells were treated with mild ER stress (0.5μM tunicamycin), and splicing levels (sXbp1) were measured at 2, 4, 6 and 18 h time points by qRT–PCR analysis (mean±s.e.m., *n*=3). The fold change in splicing levels of wild-type and triple mutant Ire1 were compared with empty vector splicing. (**b**) Western blot using an anti-sXbp1 antibody showing spliced Xbp1 protein expression levels under mild ER stress at 2, 4, 6, 18 h time points. Triple mutant shows reduced spliced Xbp1 protein expression levels as compared with wild type in Ire1−/− cells, which become less pronounced over time. (**c**) Ire1−/− cells were treated with 0.5μM tunicamycin for 6 h, after which mRNA levels of *RIDD* target genes were measured by qRT–PCR and normalized to GAPDH (mean±s.e.m., *n*=3). mRNA levels of *RIDD* target genes were lower in cells transfected with WT Ire1 relative to activation loop mutant Ire1.

To further emphasize the importance of the activation loop phosphorylations, we conducted a splicing time course experiment using Ire1−/− MEF cells in mild ER stress conditions of 0.5μM tunicamycin with triple activation loop mutant S724A, S726A, S729A and wild-type Ire1 and observed the fold change in splicing compared with empty vector by qRT–PCR analysis (Fig. 5a). We see at each time point a reduced level of splicing for the triple activation loop mutant as compared with wild type. At 2 h, the mutant level of splicing is comparable to empty vector splicing but increases twofold above empty vector before dropping back after 18 h. The wild type displays significantly more splicing at each time point with an almost 3.5-fold increase at 6 h before dropping back at 18 h. Moreover, the splicing for wild type fluctuates more than the mutant. This result clearly shows that mutation of active site phosphorylations reduces splicing in mild ER stress conditions in Ire1−/− cells and is consistent with both the HT1080 cell line and *in vitro* splicing data. Furthermore, fluctuations in wild-type splicing suggest that wild-type Ire1 may be able to adapt its splicing requirements better than mutant protein, again suggesting that active site phophorylations have a role in regulating splicing output. To investigate the effects of activation loop phosphorylation mutant upon expressed protein levels, we performed a western blot analysis for spliced Xbp1 from Ire1−/− cells at varying time points. (Fig. 5b, and Supplementary Fig. 6). We clearly see a difference between wild-type and mutant samples for spliced Xbp1 protein expression levels at all time points tested using mild ER stress, with the largest difference in protein levels occurring at a 4-h time point. The difference in spliced Xbp1 expression levels between triple mutant and wild type becomes much less pronounced during the course of the experiment, suggesting that the rate of activation is compromised more severely in the mutant protein during early stages of ER stress.

Taken together, the experiments conducted both in HT1080 and Ire1−/− cells reinforce the notion that activation loop phosphorylations are coupled to RNase activity as evidenced by both reduced mRNA splicing and reduced sXbp1 protein expression levels.

### Activation loop phosphorylation mutant reduces RIDD activity

To understand whether active site phosphorylations have an impact upon RIDD, we measured the relative mRNA levels of *RIDD* target genes^21^ in Ire1−/− cells that have been transfected either with wild-type Ire1 or with triple activation loop mutants using qRT–PCR. After 6 h of treatment with tunicamycin, we observed that five out of the six *RIDD* genes had lower mRNA levels in wild-type Ire1 samples as compared with mutant samples with *Scara3*, *Pdgfrb* and *Hgsnat* displaying almost 20% reduction in wild type compared with mutant (Fig. 5c). This suggests that RIDD activity is higher in wild-type than in mutant samples consistent with the notion that Ire1 active site phosphorylation mutants reduce RNase activity. Thus, mutation of Ire1 activation loop phosphorylations reduces Ire1’s RIDD activity and suggests that phosphorylation may have a regulatory role in RIDD activity.

## Discussion

In this study, we identify specific phosphorylations upon Ire1 that fall into three regions; linker, activation loop and RNase domain. We show that activation loop phosphorylations are important for achieving high levels of splicing *in vitro* and that mutation of activation loop phosphorylations retards splicing both in HT1080 and in Ire1−/− cells. Moreover, we show that these activation loop phosphorylations have an effect on Ire1’s RIDD activity. Thus, the data clearly link the phosphorylation events on ser724, ser726 and ser729 within the activation loop to enhanced RNase splicing and imply that kinase activation loop phosphorylations are an important and necessary step to achieve the activated Ire1 RNase state leading to enhanced Xbp1 splicing and subsequent UPR activation.

The precise mechanism of how phosphorylations activate the RNA splicing inferred from this study, and in analogy to other kinases^22^, is most likely based on conformational changes that take place within the protein. In general kinase biology^22^, the phosphorylation event causes movements within the active site that are transmitted to the rest of the protein, manifesting in gross movements of C-lobe relative to N-lobe. In a similar manner, it is most likely that Ire1 activation loop phosphorylation leads to subtle movements within the kinase active site that are transmitted to the RNase active site by conformational movements via the C-lobe of the kinase domain. These conformational changes would induce the binding of RNA substrate in a more efficient manner leading to increased splicing.

Interestingly, we see effects on splicing activity that are consistent between both *in vitro* and *in vivo* systems; however, the scale varies. This is most likely due to different rate limiting steps. The rate limiting step *in vitro* may be the conformational changes relating to association and dissociation of the RNA substrate, whereas *in vivo* the RNase domain may require other protein or cellular factors to regulate such events. This may cause differences between observations *in vitro* and *in vivo*, and such effects cannot be ruled out in this study.

To obtain the differently phosphorylated Ire1 protein for our *in vitro* analysis, we have made use of eukaryotic insect expression system that can authentically process post-translational modifications, in part, due to the presence of phosphatases, and that allows us to isolate distinct phosphorylated species. The ability to purify these distinct populations suggests that they are in a biologically relevant state. Interestingly, we see no further phosphorylation species above those seen in PK4 and no evidence of hyperphosphorylation similar to that seen in yeast Ire1 expressed in *E.coli*. A likely explanation for this is that yeast Ire1 has an extra loop insertion rich in ser and thr residues compared with the human sequence, which could be the site for extra hyperphosphorylations; alternatively, a recent report suggests a non-physiological role for hyperphosphorylation for proteins expressed in *E.coli*^23^.

**Figure 6:**
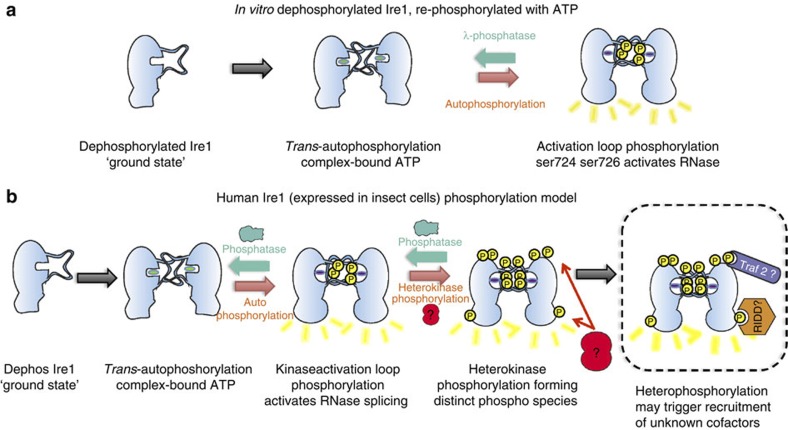
Model of phosphoregulated Ire1 activation. (**a**) *In vitro* dephosphorylated Ire1, re-phoshorylated with ATP. Depicts the situation when purified dephosphorylated human Ire1 protein (PK1) is auto re-phosphorylated *in vitro* with incubation of ATP only. Initially, the dephosphorylated protein exists in a ground state and forms a face-to-face dimer. Upon addition of ATP, trans-phosphorylation occurs on positions ser724 and ser726 of the activation loop, which causes conformation changes within the kinase domain that translate to the RNase domain resulting in activation of RNase such that splicing turnover is enhanced over 60-fold higher than that of the ‘ground state’. Ire1 then probably reorientates to form a back-to-back arrangement. (**b**) Recombinant human Ire1 expressed in insect cell model. Initially, Ire1 is dephosphorylated and in the ‘ground state’ where it forms the trans-autophosphorylation face-to-face dimer. Upon addition of ATP, autophosphorylation occurs on the activation loop resulting in pSer724, pSer726. Another factor may cause phosphorylation at other sites within the linker region and RNase domain. Phosphatases within eukaryotic cells keep phosphorylated Ire1 in a biologically relevant state. The order of phosphorylation is not known, but the final phosphorylation on the activation loop pSer729 results in activated RNase splicing such that the turnover is enhanced at least 100-fold above ground state. Phosphorylations within the RNase domain and linker region have no/little direct impact on splicing but may be recruiting factors for other process such as RIDD or Traf2; this part of the model is highlighted with dashed lines to indicate that it is speculative. At this stage, Ire1 probably reorientates to form a back-to-back arrangement.

Although the role of activation loop phosphorylations ser724, ser726 and ser729 is clearly linked to enhanced splicing, the remaining phosphorylations, notably the linker region (L-phos) and RNase domain thr973, have minimal impact on splicing. In the case of L-phos, it is too far way from the kinase or RNase active site to cause an effect. It has be suggested that maybe L-phos has a role in increasing the oligomeric state of Ire1; however, we see no biophysical evidence that any phosphorylated species exists higher than that of a dimer (Prischi *et al*. in preparation), and even if it did increase the oligomeric state we see no increase in enzymatic rates. Similarly, L-phos could cause the break-up of a dimer, but again this is unlikely as we see no corresponding loss in enzymatic rate when compared with dephosphorylated protein. Interestingly, when we allow the auto-transphosphorylation reaction to occur with dephosphorylated protein *in vitro*, we only measure ser724 and ser726 phosphorylation. The mechanism of activation loop phosphorylation whether *in vitro* or in cells can easily be rationalized by the human Ire1 crystal structure, which captures the autophosphorylation event and shows the ordered region of the activation loop protruding outward towards the kinase active site of the opposite monomer. However, there must be some cellular factor causing the phosphorylation of ser729, linker region and thr973, possibly another phosphorylating kinase. For ser729, the phosphorylation of which causes an increase in splicing, this could act as another level of regulatory control whereby initial cleavage of Xbp1 causes a tempered response to unfolded protein, but then Ire1 has another ‘gear’ so as to amplify the response. Very low levels of thr973 phosphorylation were detected and only when the Ire1 protein had already achieved high degree of phosphorylation (PK3, PK4). thr973 does not have an impact directly on the splicing activity *in vitro*, but has a small noticeable effect *in vivo*. Its position within the RNase binding domain suggests that it may be able to influence the reaction in other ways. It is tempting to speculate that this phosphorylation and the phophorylations on the linker region could act as recruitment factors for Traf2 binding and initiate apoptosis (Fig. 6a,b). Interestingly, there is now accumulating evidence to suggest that there are a number of factors that can influence the duration and amplitude of Ire1 signalling via a protein-signalling platform termed UPRosome^24^,^25^. One such example is the regulation by the BAX-BAK protein complex, which influences Ire1 signalling by helping to alleviate stress in the first instance, but if the stress signal is not relived then this complex switches to outputting an apoptotic response^24^. It may be that phosphorylations are a key component of initiating interactions with cofactors that constitute the UPRosome, and hence the importance of the present study is in trying to shed light on this interesting type of regulation.

It is clear that phosphorylation has an important role in regulating Ire1 activity and what is understood from this study is the impact that specific phosphorylations have on RNase splicing and RIDD activity and the requirement of kinase loop phosphorylations to achieve the activated Ire1 RNase splicing state for UPR signal activation.

## Methods

### Expression and purification of phosphorylated Ire1 species

The C-terminal domain of human Ire1α (residues 547–977) was expressed in sf9 insect cells using the Bac-to-Bac method after a 3-day-growth at 27 °C (ref. 3). The cell pellet was lysed by sonication in 50 mM Hepes, pH 7.5, 300 mM NaCl, 10% glycerol, supplemented with protease inhibitors and centrifuged to remove cell debris and insoluble material at 16,000 *g* for 60 min. The supernatant was then passed through a batch/gravity column containing 15 ml of talon resin (Clontech), and protein was eluted off with 300 mM imidazole in a total volume of 50 ml^3^. Sample was treated with Rhinovirus 3C protease (PreScission protease, Amersham Biosciences, 300 ml at 2 mg ml^−1^) to remove his-tag. To obtain distinct Ire1 phosphorylated species, the lambda phosphatase incubation step was omitted^3^ and sample was passed through Mono-Q column with a shallower gradient (80–300 mM NaCl over 30 column volumes). The eluted phosphorylated peaks were separated and further purified by size exclusion chromatography on a Superdex200 column (GE Healthcare) in a 25 mM Hepes pH 7.5 buffer, containing 150 mM NaCl, 5 mM DTT and 5% glycerol.

### *In vitro* autophosphorylation

Ire1 autophosphorylation was achieved by incubation for at least 2 h of 50 μM PK1/dephosphorylated Ire1 with 2 mM ATP (Sigma), 5 mM MgCl_2_, at room temperature in a 25 mM HEPES, pH 7.5, containing 150 mM NaCl, 5 mM DTT and 5% glycerol. The presence of autophosphorylation was confirmed by mass spectrometry and western blot using a pSer724 phospho-specific antibody.

### *In vitro* RNase splicing assay

Ire1α RNase activity was measured by incubation of 0.2 μM purified protein with increasing concentrations (0–50 μM) of quenched single-strand RNA probe (5′- Cy3-GACGUCCACAUCCUGGUCC-BHQ2 -3′ IDT DNA Technologies, Leuven, Belgium) at 30 °C in a 384-well low-volume non-binding plate (Greiner Bio-One Ltd, Gloucestershire, UK) with a final reaction volume of 20 μl. Ire1α reaction buffer (25 mM HEPES pH 7.5, 150 mM NaCl, 5 mM DTT, 5% glycerol) was used for all RNase activity measurements. Whether in presence of ATP or ADP or AMP-PNP (Sigma) the same protocol of the autophosphorylation was used (incubation of 50 μM protein for at least 2 h with 2 mM nucleotide and 5 mM MgCl_2_). Time-dependent fluorescence was measured on a POLARstar Omega plate reader (BMG LABTECH GmbH, Germany) using an excitation filter of 550 nm and emission filter of 590 nm. Initial rates were measured using MARS Data Analysis Software V2.41 (BMG LABTECH GmbH, Germany). Initial rates were plotted with a Michaelis and Menten model using PRISM 6 (GraphPad Software, La Jolla California USA, www.graphpad.com).

### Cell culture and *in vivo* Xbp1 mRNA splicing assays

Human Fibrosarcoma HT1080 cells were cultured in Dulbecco’s Modified Eagle Medium supplemented with 10% Fetal Bovine Serum, 2 mM L-Glutamine, 50U Penicillin per 50 μg Streptomycin per ml. A day before transfection, 300,000 cells per well (2 ml) were plated on a six-well plate. Wells were transfected with 2 μg of DNA mixed with Fugene 6 reagent (Promega) in a 1:3ratio.2 μg of total DNA per well were prepared by mixing equimolar ratio (1:1) of Wild-Type/Mutants IRE1α (Adgene pcDNA3 plasmid) and Adgene’s pEGFP-C3 (transfection efficiency indicator). After 24 h, IRE1α- expressing cells in each well were induced by 0.5 μM Tunicamycin dissolved in DMSO (0.5% v/v) and harvested after 2, 4 and 6 h. The total RNA was extracted from the cells by using RNeasy Plus Mini Kit (Qiagen), and were DNAse I treated (1U/10 μl RNA) and reverse transcribed to cDNA by SuperScript III First-Strand Synthesis SuperMix (by random hexamers). The spliced (228 bp) and unspliced (254 bp) Xbp1 cDNA fragments were amplified by PCR (Phusion Flash High Fidelity PCR Master Mix-Thermo Scientific) using 68 °C as an annealing temperature and following set of primers: foward primer: 5′-CCTGGTTGCTGAAGAGGAGG-3′ and reverse primer: 5′-CCATGGGGAGATGTTCTGGAG-3′. PCR products were run on 3% agarose gel stained with Gel Red dye (Biotium) in 1 × TBE buffer. All samples were made in triplicate and subjected to densitometry measurements by Phoretix gel analysis software (TotalLab). Splicing percentage values are shown as an average value of three measurements±s.d. The same experiments were performed in Ire1−/− cells. Ire1−/− MEFs (kindly provided by Professor David Ron) were grown in Dulbecco’s Modified Eagle Medium (high glucose) supplemented with 10% Fetal Bovine Serum, 2 mM L-glutamine, 1 × non-essential amino-acid solution, 50 μM 2-Mercaptoethanol and 50U Penicillin per 50 μg Streptomycin per ml. A total of 500,000 cells were plated 24 h before transfection, then transfected with 2 μg DNA (equimolar mixture of plasmids+pEGFP-C3) in ratio 1:6 with Fugene 6 reagent (Promega). After 48 h (peak of IRE1a expression), cells were induced with 5 μM tunicamycin and harvested, respectively, after 2, 4 and 6 h. Further stages of experiment were performed as described previously.

### Quantitative RT–PCR

MEF Ire1−/− retrotranscribed cDNA was used in triplicate for quantitative real-time PCR analysis using the SYBR Green reagent system (Applied Biosystems) and an ABI 7500 (Applied Biosystems). Relative quantities of amplified cDNAs were then determined using SDS v 1.4 software and normalized to GAPDH.

### Western blot analysis

Induced MEFs in each well of asix-well plate containing IRE1a−/− cell monolayer were lysed in RIPA buffer, scraped and agitated for 15 min at 4 °C. After that cell extracts were incubated on ice for 30 min and centrifuged for 10 min at 10,000 *g*. Collected supernatants were mixed with Leammli buffer and run on 4–12% precast Bis-Tris gels (Invitrogen). Gels were transferred to nitrocellulose membrane (Invitrogen’s iBlot) and blocked in PBS+0.05% Tween-20 and 5% Marvel Dried Milk. Primary antibody was added to blocking buffer (PBS, Tween-20, 2.5% milk powder) in concentration of 1:200 for antispliced human Xbp1s (Biolegend) and 1:2,000 for antihuman GAPDH FL-335 (Santa Cruz Biotechnology)(Full image western blot of sXbp1 over time in Ire1−/− cells is presented in Supplementary Fig. 7). After a 1 h incubation at room temperature, membranes were washed three times in PBS+0.05% Tween-20 and incubated with secondary antibody (anti rabbit, GE Healthcare) diluted (1:2,000) in 2.5% milk blocking buffer (1 h, room temperature). Followed by another three washes, blots were visualized by Millipore Luminata Crescendo Western HRP substrate and developed on Amersham Hyperfilm ECL (GE Healthcare).

### Statistical methods

All experiments were performed in triplicate unless otherwise stated.

### Mass spectrometry

*MS MALDI TOF*. Mass spectrometry. In preparation for mass spectrometry, the desired protein band was excised, lyophilized and digested with trypsin (E.C.3.4.21.4, Promega) overnight. Peptides were extracted from gel pieces, and nanoLC was performed on an Ultimate 3000 using a PepMap 100 75 mm × 15-cm-fused silica C18 analytical column (LC Packings, Dionex, Sunnyvale, CA), coupled to a Probot for fraction collection and matrix addition with α-Cyano-4-hydroxycinnamic acid acid as the matrix. A gradient of 2–60% ACN in 0.1% TFA was delivered over 36 min at a flow rate of 0.300 nl min^−1^. MALDI TOF/TOF-MS was performed using an Applied Biosystems 4800 mass spectrometer (Foster City, CA.) in the positive reflectron mode with delayed extraction. MS precursor acquisition was followed by interpretation and data-dependent MS/MS acquisition with the CID on. Data interpretation was configured to select a maximum of 10 precursor ions per fraction with a minimum signal-to-noise ratio of 50. The data were processed using GPS Explorer (Applied Biosystems, CA) against the Swiss-Prot database. Search parameters were enzyme=trypsin; fixed modifications=carboxymethyl (C); variable modifications=oxidation (M); mass tolerance±100 p.p.m.; fragment mass tolerance=0.3 Da; maximum missed cleavages=1; mass values=monoisotopic. Phosphorlyated peptides were assigned manually on inspection of the data.

### Mass analysis of intact protein

Samples were desalted by adsorption to Millipore C4 Ziptip, washed with 5%(v/v) acetic acid and eluted to the sample slide with 2 μl of CHCA matrix (10 mg ml^−1^ alphacyano-4-hydroxycinnamic acid in 50% acetonitrile/water conatining 0.1%trifluroacetic acid). After drying at room temperature, samples were analysed in a Waters MaldiMicro MX mass spectrometer operating in linear mode with a 10 Hz UV laser, power 130, pulse voltage 850 V, ion extraction delay time 2,000 ns with external calibration using multiple charge states of bovine trypsinogen and its dimer. Spectra were displayed as the average of ~1,000 laser shots each. Data processing was performed by using Waters MassLynx software.

*ESI QTOF*. Sample processing. For intact molecular mass determination, samples were button dialysed (Hapton Research, Aliso Viejo, CA, USA) overnight into 50 mM ammonium acetate, pH 6.9. For phosphorylation site analysis, samples were separated by 1D SDS-PAGE. The phosphoprotein band was excised for proteolytic digestion with trypsin followed by phosphopeptide enrichment by TiO_2_ chromatography. In brief, 2.5 μg of each protein was mixed 1:1:1 (v/v/v) with 3 × Tris sample buffer and 150 mM DTT and heated at 95 °C for 5 min. Samples were cooled, centrifuged and loaded onto 15% Tris-glycine gels. A constant voltage of 200 V was applied for 1.5 h (running buffer 1 × SDS tris/glycine). The gels were rinsed with distilled water (5 min) and then stained with InstantBlue Coomassie stain (Expedeon Hartson, UK) overnight. Phopshoprotein gel bands were excised and chopped into small pieces (~1 mm^3^ ) and destained in 30% ethanol at 70 °C for 30 min with shaking. This was repeated with fresh ethanol solution until all coomassie stain was removed. The gel was then covered with 25 mM ammonium bicarbonate/50% acetonitrile and vortexed for 10 min. The gel slices were then covered with 100% acetonitrile and left for 5 min with vortexing before the supernatant was discarded and replaced with a fresh aliquot of acetonitrile. Acetonitrile was removed, and the gel pieces were completely dried under vacuum centrifugation for 30 min. Once dry, the gel slices were cooled on ice and then covered with ice cold trypsin solution (20 ng μl^−1^ in 25 mM ammonium bicarbonate) and left on ice for 30 min to rehydrate. Excess trypsin solution was removed, and the gel slices were covered with a minimal amount of 25 mM ammonium bicarbonate. After briefly vortexing and centrifuging, the gel slices were incubated at 37 °C with shaking for 18 h. The resulting digest was vortexed, centrifuged and 50 μl water was added. Following vortexing for 10 min, the supernatant was recovered and added to an eppendorf containing 5 μl acetonitrile/water/formic acid (60/35/5; v/v). Fifty microlitres of acetonitrile/water/formic acid (60/35/5; v/v) was added to the gel slices and vortexed for an additional 10 min. The supernatant was pooled with the previous wash, and one additional wash of the gel slices was performed. The pooled washes containing the peptides were dried by vacuum centrifugation. For phosphopeptide enrichment by TiO_2_ affinity chromatography, the tryptic digests were reconstituted in 25 μl loading buffer (1 M glycolic acid (Sigma Aldrich, UK) in 80% acetonitrile/5% TFA). A TiO_2_ micro-column was prepared by plugging the constricted end of a 200 μl GELoader pipette tip with a C8 disc. TiO_2_ beads (GL Sciences, Japan) were suspended in 100% acetonitrile and packed to column length of 3 mm by the application of gentle air pressure. The column was flushed with 50 μl of 50% acetonitrile and equilibrated with 20 μl of loading buffer. The peptide solution was then passed through the column and the flow-through collected. The column was washed with 5 μl of loading buffer followed by 30 μl of washing buffer (80% acetonitrile/1% TFA) ,and the eluates were pooled with the flow-through. Bound peptides were eluted from the column with 50 μl elution buffer (0.5% ammonia solution) followed by 1 μl of 30% acetonitrile. All fractions were lyophilized to complete dryness and stored at −20 °C until ready for MS analysis.

Enriched samples were reconstituted in 10 μl water and desalted using ZipTip C18 tips (Millipore UK Ltd, Watford, UK) into 50% acetonitrile and analysed by Z-spray nanoelectrospray ionization MS using a quadrupole-IMS-orthogonal time-of-flight MS (Synapt HDMS, Waters UK Ltd., Manchester, UK) using gold/palladium-coated nanospray tips. The MS was operated in positive TOF mode using a capillary voltage of 1.5 kV, cone voltage of 20 V, nanoelectrospray nitrogen gas pressure of 0.1 bar, backing pressure of 2.47 mbar and collision energy of 15–25 V in the trap. The source and desolvation temperatures were set at 80 and 150 °C, respectively. During TOF-MS acquisition, Argon was used as the buffer gas, at a pressure of 4.0 × 10^−3^ mbar in the trap and transfer. Mass calibration was performed by a separate injection of sodium iodide at a concentration of 2 μg μl^−1^. Data processing was performed using the MassLynx v4.1 suite of software supplied with the mass spectrometer.

## Author contributions

F.P. isolated and purified different phosphorylation species, designed, performed and analysed FRET data, performed and analyzed qRT-PCR data, analysed Mass spec and cloned some *in vivo* mutants. P.R.N. expressed constructs in sf9 cells, cloned Ire1 mutants, performed and analysed Ire1 *in vivo* RT–PCR splicing experiment and maintained all cell cultures. M.C. helped with cloning and purification of *in vivo* mutants. M.M. U.A. conceived, designed and supervised experiments and wrote the manuscript.

## Additional information

**How to cite this article:** Prischi, F. *et al*. Phosphoregulation of Ire1 RNase splicing activity. *Nat. Commun.* 5:3554 doi: 10.1038/ncomms4554 (2014).

## Supplementary Material

Supplementary Information Supplementary Figures 1-7, Supplementary Table 1 and Supplementary Note 1

## Figures and Tables

**Table 1:** Enzymatic rates of RNase splicing; the kinetic parameters of various dephosphorylated and phosphorylated states of Ire1.

	***V***_**max**_ **(μM s** ^**−1**^ **)**	***K***_**m**_ **(M)**	***K***_**cat**_ **(s** ^**−1**^ **)**	***K***_**cat**_**/*****K***_**m**_ **(M**^**−1**^ **s**^**−1**^**)**	**(** ***K***_**cat**_ **)**_**sample**_ **/(** ***K***_**cat**_ **)**_**dephos**_	**(** ***K***_**cat**_ **/** ***K***_**m**_ **)**_**sample**_ **/(** ***K***_**cat**_ **/** ***K***_**m**_ **)**_**dephos**_
dephos Peak 1	0.024	3.68 × 10^−6^	0.118	3.21 × 10^4^	1.0	1.0
dephos+ADP	0.059	6.40 × 10^−6^	0.295	4.61 × 10^4^	2.5	1.4
dephos+AMP-PNP	0.029	5.48 × 10^−6^	0.144	2.63 × 10^4^	1.2	0.8
dephos+ATP (*in vitro* re-phos)	1.36	1.62 × 10^−5^	6.79	4.20 × 10^5^	57.5	13.0
Peak 2	0.28	2.78 × 10^−5^	1.42	5.11 × 10^4^	12.0↓ × 5	1.6
Peak 3	1.47	2.44 × 10^−5^	7.37	3.02 × 10^5^	62.4↓ × 2	9.4
Peak 4	2.5	9.95 × 10^−6^	12.5	1.26 × 10^6^	105.9	39.2
