# Supplementary material for: Phosphoregulation of Ire1 RNase splicing activity
Source: Nat Commun. 2014 Apr 7;5:3554. doi: 10.1038/ncomms4554 (PMC3988810; doi:10.1038/ncomms4554)
Supplement: Supplementary Information — Supplementary Figures 1-7, Supplementary Table 1 and Supplementary Note 1 [file ncomms4554-s1.pdf]

Supplementary Fig. 1A)

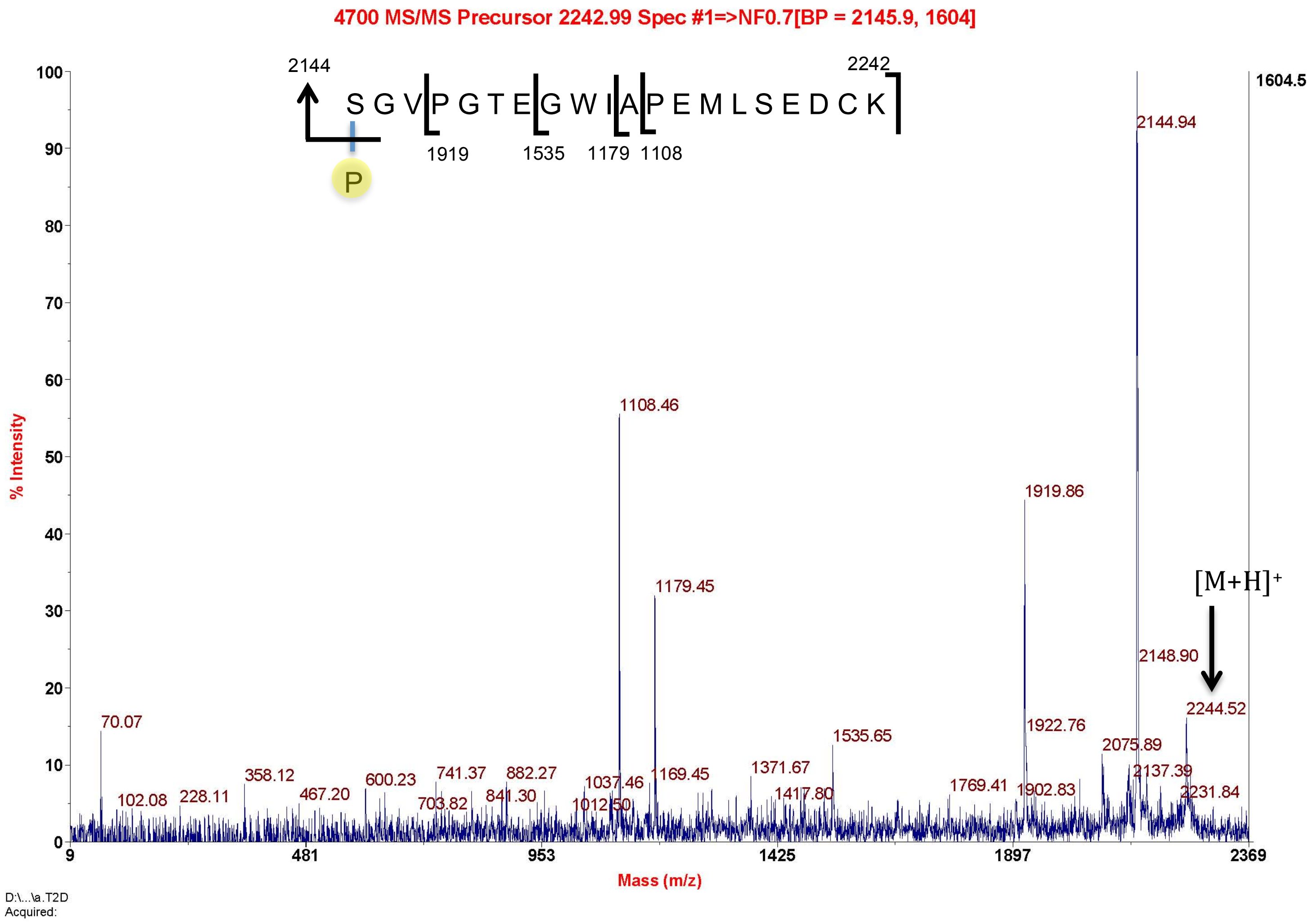

1B)

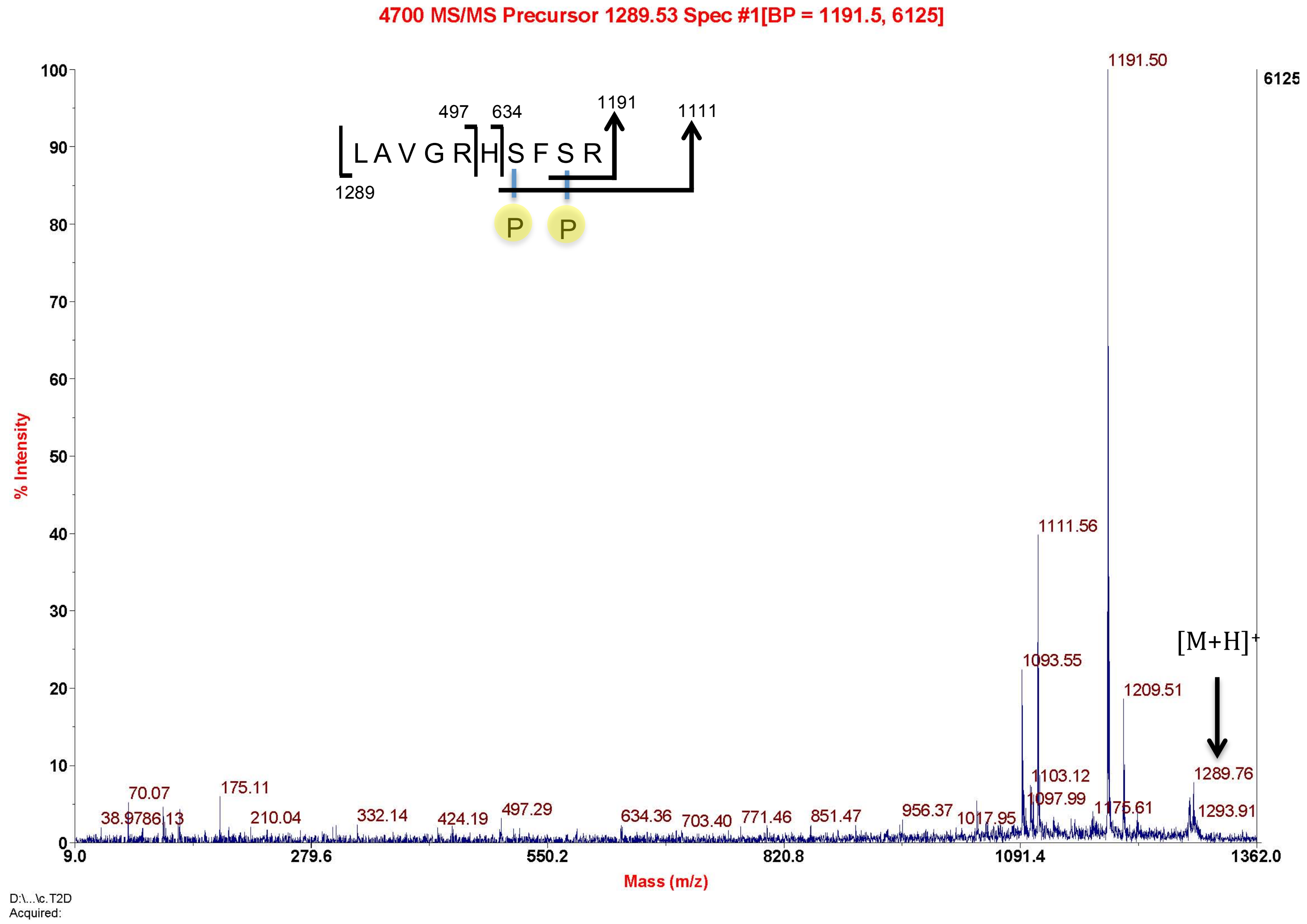
4700 MS/MS Precursor 1289.53 Spec #1[BP = 1191.5, 6125]
LAVGRHSFSR
497
634
1191
1111
1289
P
P
[M+H]+
% Intensity
Mass (m/z)
1191.50
6125
1111.56
1093.55
1209.51
1289.76
1103.12
1097.99
1175.61
1293.91
70.07
175.11
38.97
86.13
210.04
332.14
424.19
497.29
634.36
703.40
771.46
851.47
956.37
1017.95
9.0
279.6
550.2
820.8
1091.4
1362.0
D:\...\c.T2D
Acquired:

1C)

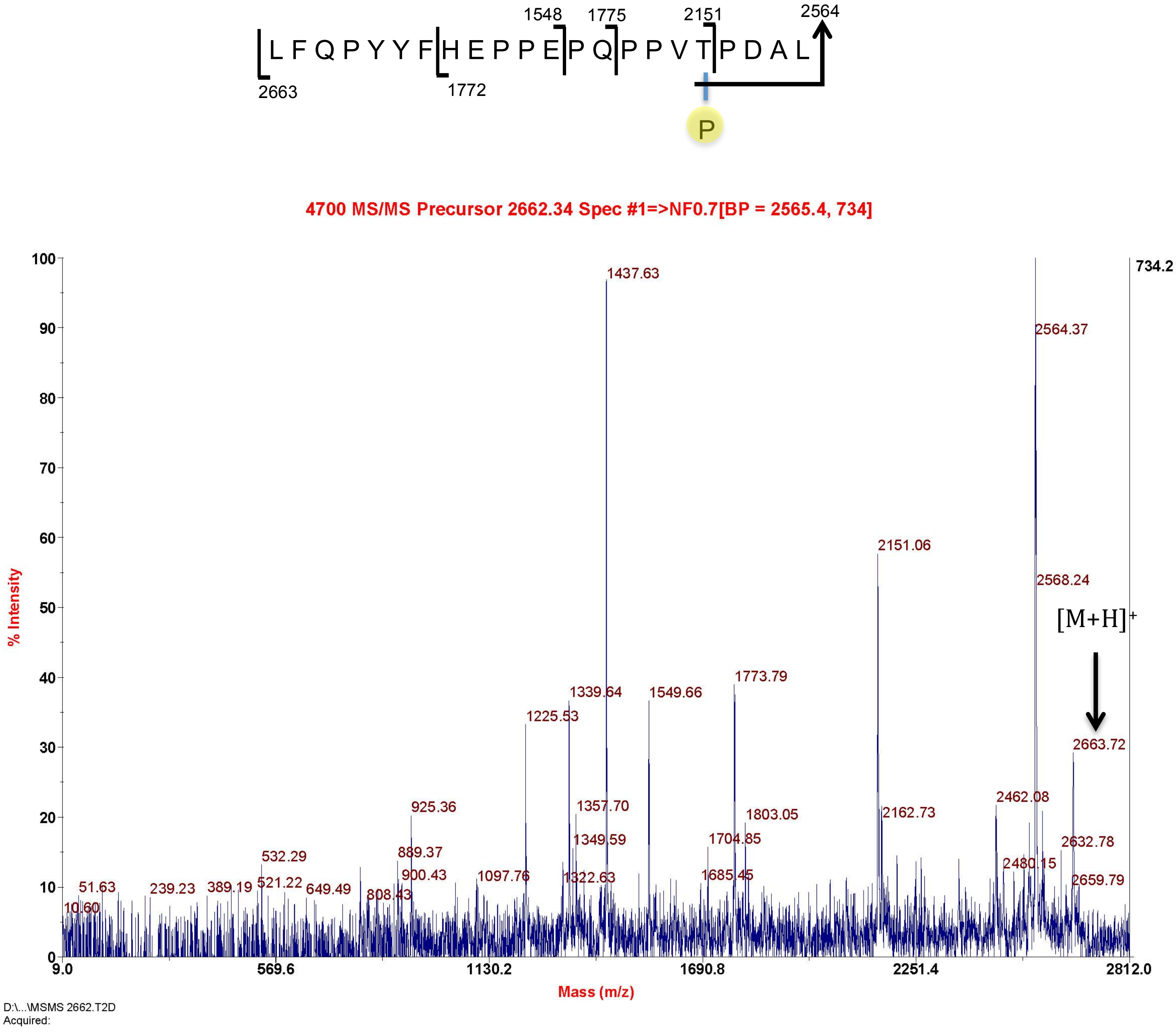

Supplementary Figure 1: Mass spectra of phosphopeptides using Maldi Tof. A) Mass spectrum of p729 phosphopeptide. **B)** Mass spectrum of p724 and p726 double phosphorylated peptide. **C)** Mass spectrum of p973 phosphopeptide**.**

Supplementary Fig. 2A)

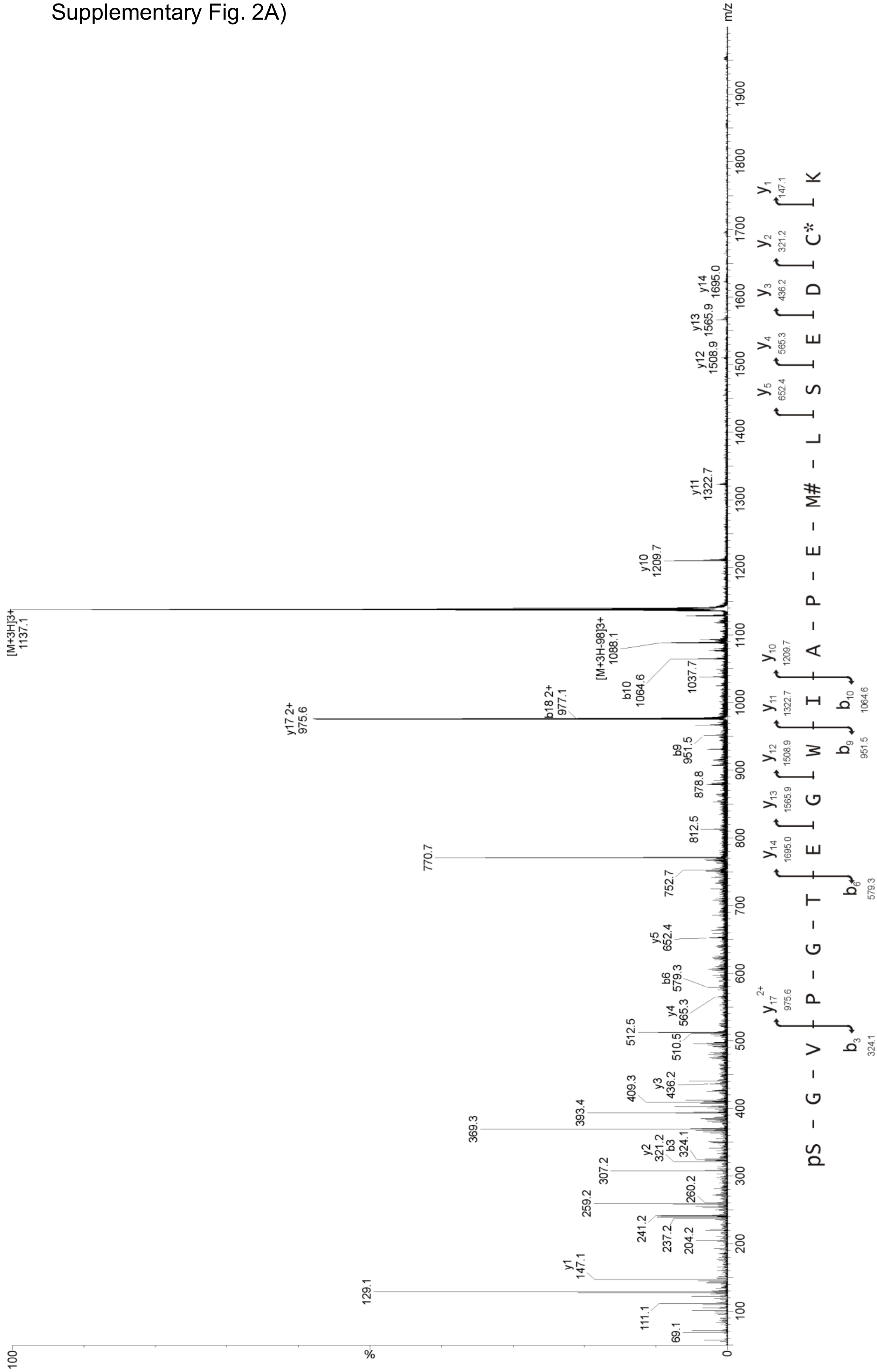

2B)

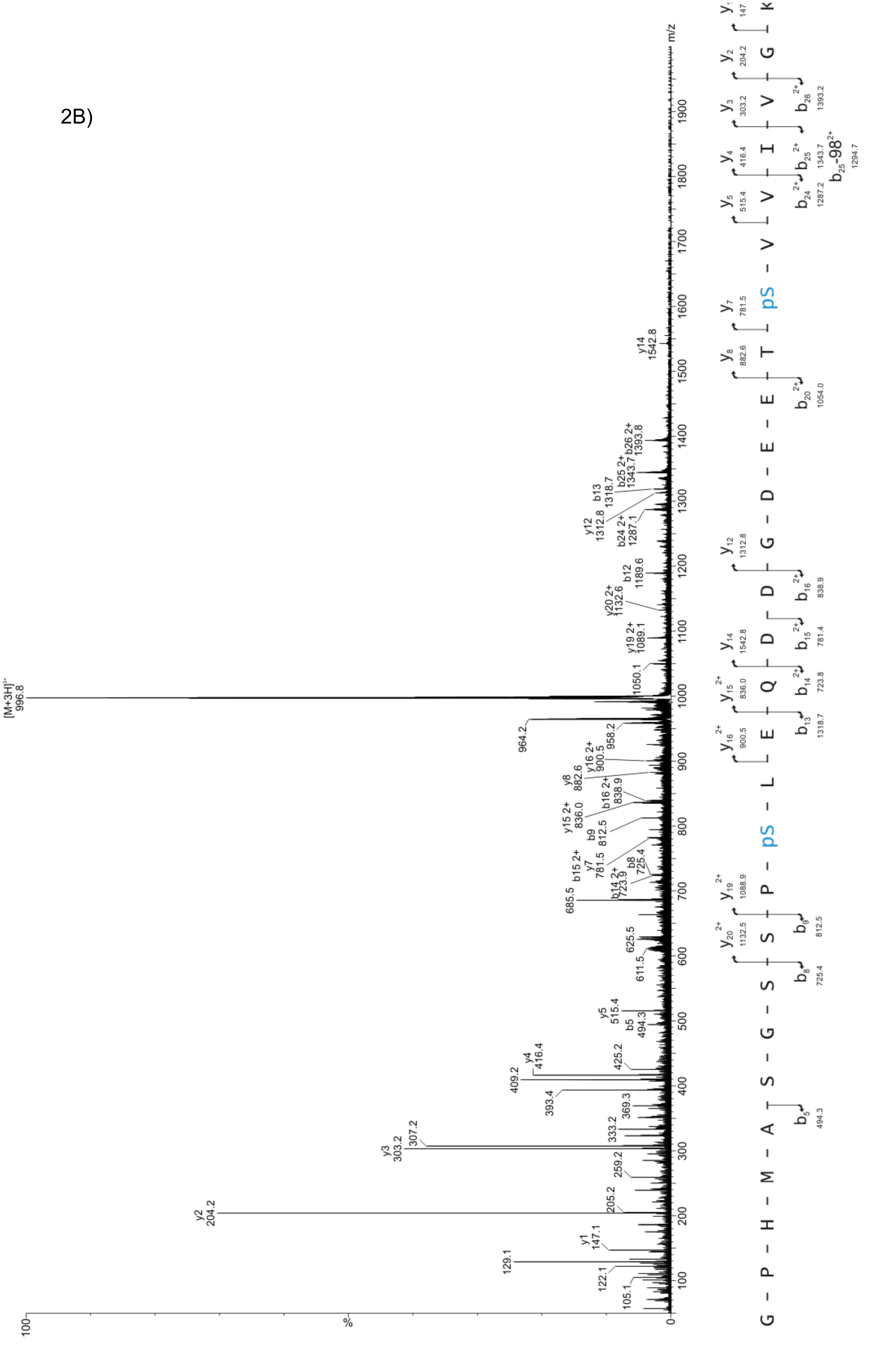
[M+3H]3+ 996.8
y2 204.2
y3 303.2
307.2
y1 147.1
129.1
122.1
105.1
205.2
259.2
333.2
369.3
393.4
409.2
y4 416.4
425.2
b5 494.3
y5 515.4
611.5
625.5
685.5
b14 2+ 723.9
b8 725.4
b15 2+ y7 781.5
b9 812.5
y15 2+ 836.0
b16 2+ 838.9
y8 882.6
y16 2+ 900.5
958.2
964.2
1050.1
y19 2+ 1089.1
y20 2+ 1132.6
b12 1189.6
y12 1312.8
b13 1318.7
b24 2+ 1287.1
b25 2+ 1343.7
b26 2+ 1393.8
y14 1542.8
%
100
0
m/z
G - P - H - M - A - S - G - S - S - P - pS - L - E - Q - D - D - D - G - D - E - E - T - pS - V - V - I - V - G - K

Supplementary Figure 2: ESI Q/TOF Mass spectra of phosphopeptides identify specific phosphorylation sites. A) ESI Q/TOF mass spectrum of p729 phosphopeptide. B) ESI Q/TOF mass spectrum of p551 and p562 containing phosphopeptide.

Supplementary Fig. 3

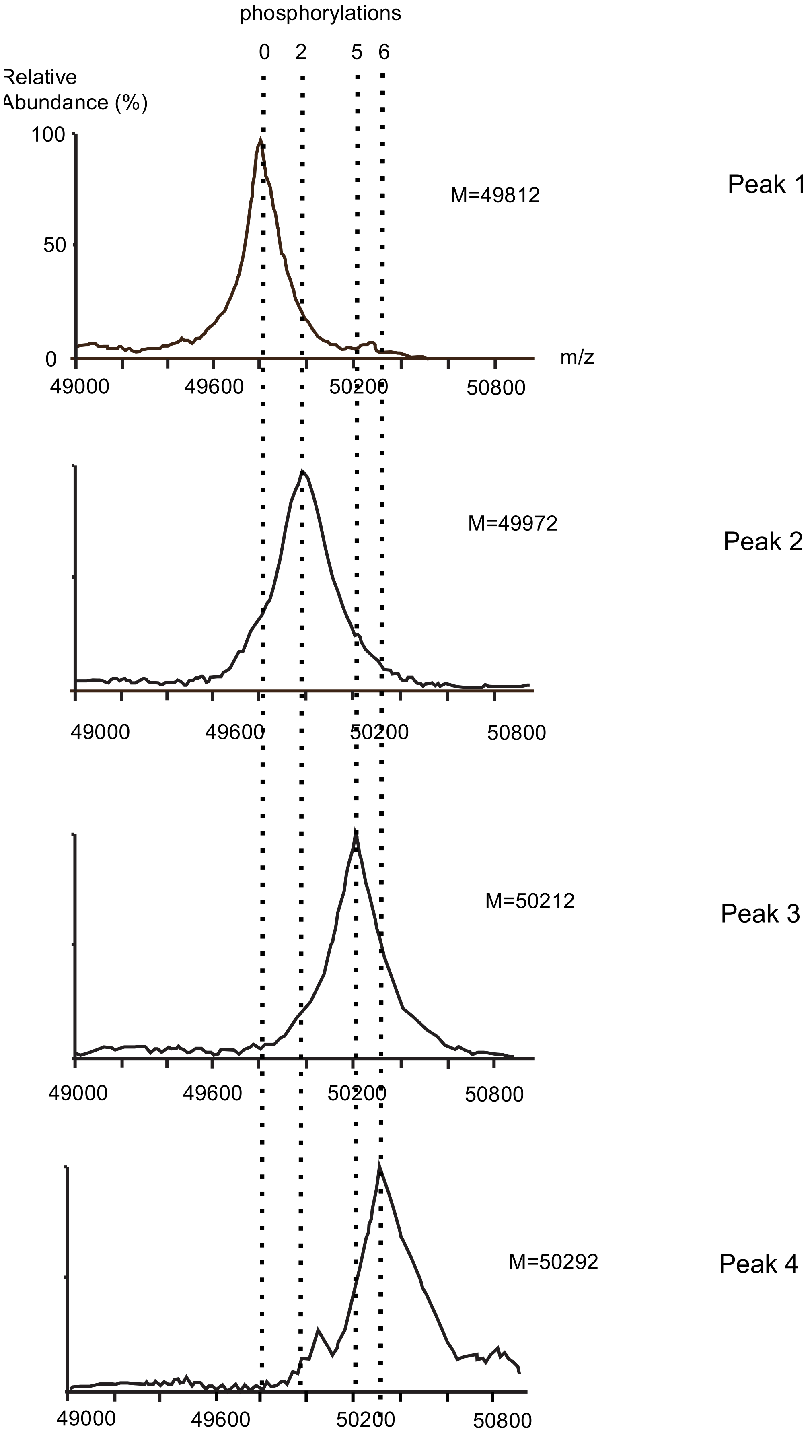

Supplementary Figure 3: Low resolution mass spectra of phosphorylated protein from elution peaks after MonoQ purification.
Low resolution Maldi TOF mass spectra of dephophorylated and phosphorylated protein from monoQ purification (peaks 1- 4, figure 1A).
Dotted lines representing the m/z values for Ire1 phosphorylated with 0, 2, 5 and 6 phosphorylations. The molecular mass of protein present within the peak sample is given along side each mass spectrum.

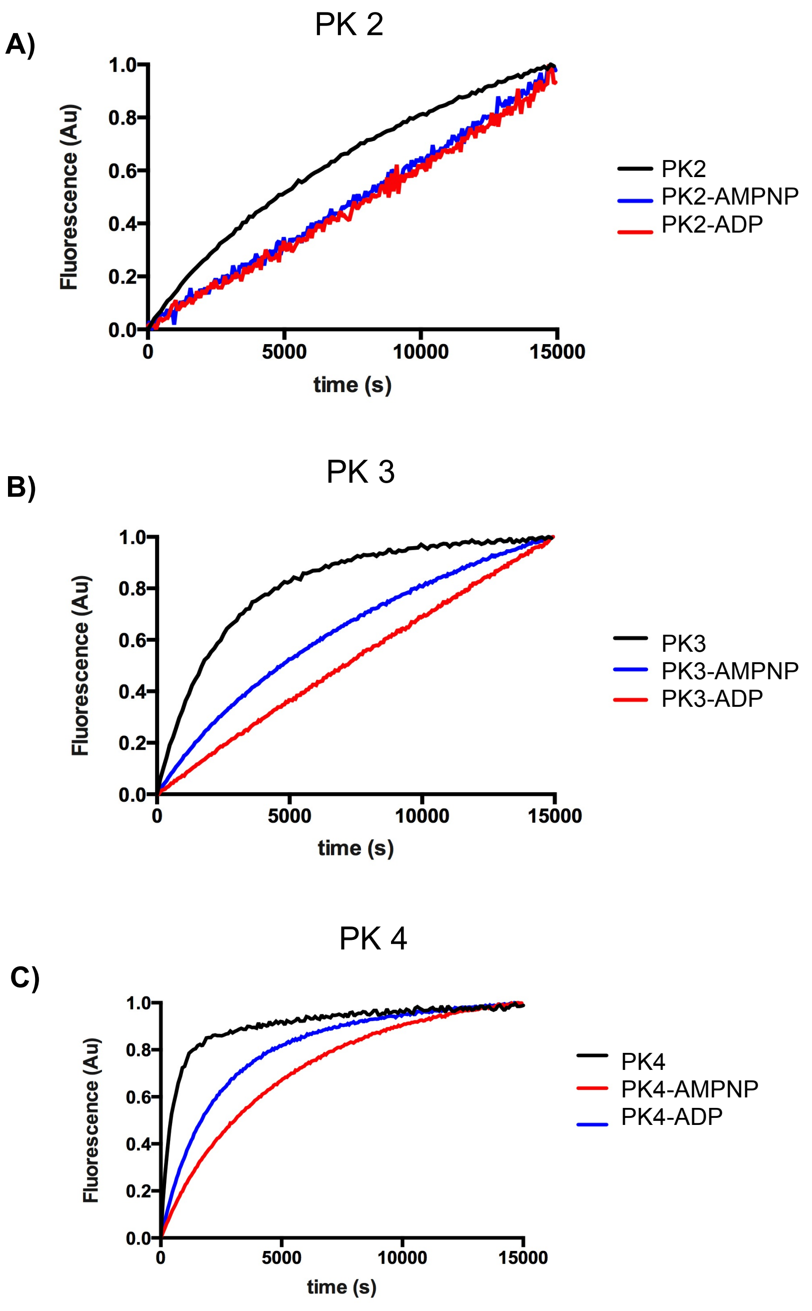

Supplementary Figure 4: Ligand binding reduces the rate of splicing of differently phosphorylated Ire1. Fluorescence time course splicing experiments with each distinct purified phosphorylated species. Ire1 apo form (black profile), Ire1 incubated with AMP-PNP (blue) and with ADP (red). The phosphorylated samples all displayed inhibition upon binding ligand with the addition ADP exhibiting the greatest inhibition of splicing. **A)** PK2 sample containing L-phos. **B)** PK3 with L-phos, pSer724, pSer726, pThr973. **C)** PK4 with L-phos, pSer724, pSer726, pSer729, pThr973. Experiments were done in triplicate.

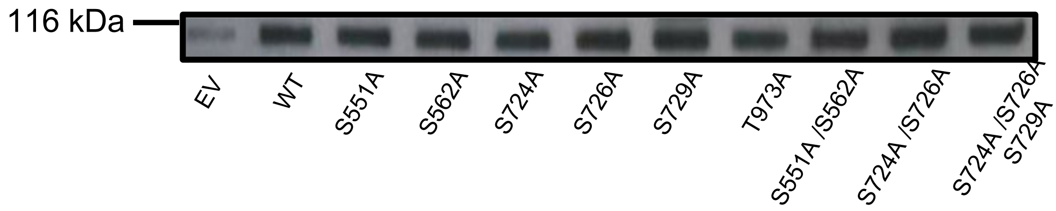

Supplementary Figure 5: Expression levels of Ire1 in HT1080 cells.
A western blot using Ire1 specific antibody showing the expression levels of transiently transfected HT1080 with plasmids containing wild type and mutant Ire1 used in this study and compared to empty vector endogenous Ire1 expression.

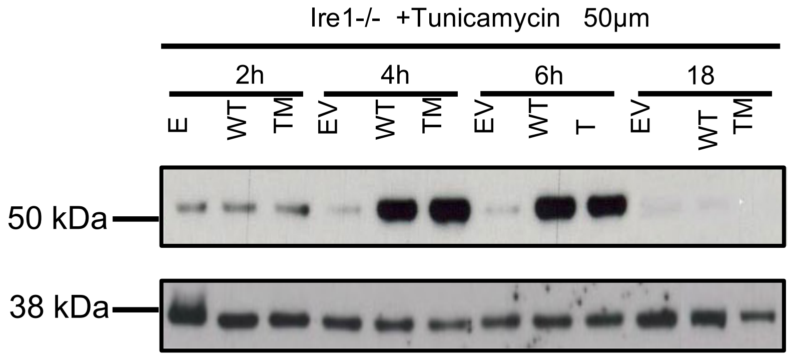

Supplementary Figure 6: Xbp1 Spliced protein expression levels in Ire1-/- cells with 50µM tunicamycin. At high concentrations of ER stress both WT and triple mutant (TM) produce elevated levels of protein as compared to 0.5**µ**M tunicamycin and thus subtle differences between WT and TM are not seen. After 18hours the cells are no longer viable. GAPDH was used as loading control.

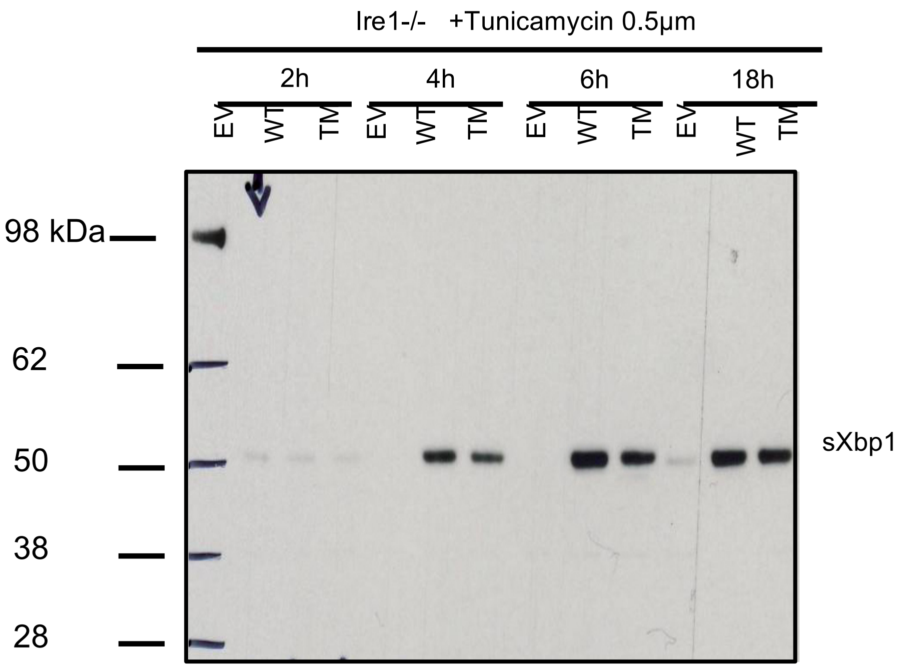

Supplementary Figure 7: Full sized image of Western blot illustrated in Figure 5B. Using anti sXbp1 antibody showing spliced sXbp1 protein expression levels under mild ER stress at 2, 4, 6, 18 hour time points. Triple mutant shows reduced spliced Xbp1 protein expression levels as compared to wildtype in Ire1 -/- cells, which becomes less pronounced over time.

Peak 1
No phosphorylated peptides detected

Peak 2
2 phosphorylations detected
G P H M A S G S S P S L E Q D D G D E E T S V V I V G K

Peak 3
5 phosphorylations

G P H M A S G S S P S L E Q D D G D E E T S V V I V G K

L A V G R H S F S R

L F Q P Y Y F H E P P E P Q P P V T P D A L (signal weak for this peptide)

Peak 4
6 phosphorylations

G P H M A S G S S P S L E Q D D G D E E T S V V I V G K*

L A V G R H S F S R

L F Q P Y Y F H E P P E P Q P P V T P D A L (signal weak for this peptide)

S G V P G T E G W I A P E M L S E D C K*

Supplementary Table 1: Identification of phosphopeptides by mass spectrometry. Peaks taken from the monoQ purification were trypsin digested and subjected to mass spectrometry using two methods, Maldi TOF/TOF and high resolution ESI Q/TOF.

Supplementary Note 1: Using the Maldi TOF we were able to obtain good quality spectra of digested fragments and were able to identify phosphorylated peptides. For peptides that had more than one possible site for phosphorylation (denoted by *) we used ESI Q/TOF to map and identify that particular site of phosphorylation.
